# EZH2, HIF-1, and Their Inhibitors: An Overview on Pediatric Cancers

**DOI:** 10.3389/fped.2018.00328

**Published:** 2018-11-19

**Authors:** Marco Papale, Elisabetta Ferretti, Giuseppe Battaglia, Diana Bellavia, Antonello Mai, Marco Tafani

**Affiliations:** ^1^Department of Experimental Medicine, Sapienza University, Rome, Italy; ^2^IRCCS Neuromed, Isernia, Italy; ^3^Department of Molecular Medicine, Sapienza University, Rome, Italy; ^4^Department of Drug Chemistry and Technologies, Sapienza University, Rome, Italy

**Keywords:** HIF-1, EZH2, tumors, cancers, oncology, hypoxia, epigenetics

## Abstract

During the past decades, several discoveries have established the role of epigenetic modifications and cellular microenvironment in tumor growth and progression. One of the main representatives concerning epigenetic modification is the polycomb group (PcG). It is composed of different highly conserved epigenetic effector proteins preserving, through several post-translational modifications of histones, the silenced state of the genes implicated in a wide range of central biological events such as development, stem cell formation, and tumor progression. Proteins of the PcG can be divided in polycomb repressive complexes (PRCs): PRC1 and PRC2. In particular, enhancer of zeste homolog 2 (EZH2), the catalytic core subunit of PRC2, acts as an epigenetic silencer of many tumor suppressor genes through the trimethylation of lysine 27 on histone H3, an essential binding site for DNA methyl transferases and histone deacetylases. A growing number of data suggests that overexpression of EZH2 associates with progression and poor outcome in a large number of cancer cases. Hypoxia inducible factor (HIF) is an important transcription factor involved in modulating cellular response to the microenvironment by promoting and regulating tumor development such as angiogenesis, inflammation, metabolic reprogramming, invasion, and metastatic fate. The HIF complex is represented by different subunits (α and β) acting together and promoting the expression of vascular endothelial growth factor (VEGF), hexokinase II (HKII), receptor for advanced glycation end products (RAGE), carbonic anhydrase (CA), etc., after binding to the hypoxia-response element (HRE) binding site on the DNA. In this review, we will try to connect these two players by detailing the following: (i) the activity and influence of these two important regulators of cancer progression in particular for what concerns pediatric tumors, (ii) the possible correlation between them, and (iii) the feasibility and efficiency to contrast them using several inhibitors.

## Introduction

During the past decades, a growing number of scientific data has led to the discovery of epigenetics, defined as the changes during the development of an organism that are not related to DNA sequences. Changes in the chromatin configuration at the gene promoter influenced by chemical modification such as acetylation, methylation, ubiquitylation, and phosphorylation are part of an exact mechanism that regulates the accessibility to DNA and the consequent transcription and expression of coding genes (1). Some of these modifications are considered inheritable in plants, invertebrates, and also vertebrates both cell division and cell generation (germline). In fact, environmental pressures as well as lifestyle can produce epigenetic changes that can be maintained through two or three generations even in the absence of the specific stimulus (2). However, epigenetic modifications, unlike genetic mutations, are reversible because they fail to alter the nucleotide sequence.

One of the most important epigenetic regulators is represented by the polycomb group (PcG) of proteins influencing the expression of many genes implicated in the development of the organism from childhood to adulthood. Moreover, the PcG represents evolutionarily conserved multiprotein repressive complexes such as polycomb repressive complex 1 (PRC1) and PRC2. In particular, histone methyltransferase enhancer of zeste homolog 2 (EZH2), a specific component of PRC2, regulates the trimethylation of Lys 27 on histone 3 (H3K27) implicated in the expression of some essential genes in the early steps of X-chromosome inactivation in women (3). Moreover, deregulation of this particular complex has been associated with a large number of tumors, and the expression of PcG proteins is closely associated with carcinogenesis (4).

The development of a tumor is a complicated process in which a large number of *influencers* play their own role. The correlation between tumors and microenvironment was hypothesized in the last century, and it was part of Stephen Paget's (1889) “seed and soil” theory Paget's 1889 “seed and soil” theory postulating that metastatic cells of a specific cancer (“the seed”) usually metastasize to some sites (“the soil”) depending on the affinity between initial and secondary tumor sites (5).

Nowadays, we know that particular conditions of the hypoxic tumor microenvironment could promote the expression of specific oncogenes resulting in the progression of cancer. One of the most important factors regulating the response of tumor cells to hypoxia is represented by hypoxia-inducible factor 1 (HIF-1) (6, 7).

Tumor environment is characterized by the lack of oxygen, and the cells are exposed to hypoxia. In this specific hypoxic “habitat,” HIF-1 is activated. Specifically, HIF-1 is a heterodimeric protein composed of two subunits: HIF-1β (constitutively expressed) and HIF-1α (O_2_-regulated). Under normoxic conditions, the newly synthesized HIF-1α is hydroxylated at proline residues 402 and/or 564 by specific prolyl hydroxylase domain (PHD) proteins (i.e., PHD2) using O_2_ and α-ketoglutarate as substrates to catalyze a dioxygenase reaction. During such a reaction, one oxygen is inserted into the proline residue while the other oxygen is inserted into α-ketoglutarate leading to the formation of succinate and CO_2_. Next, the protein OS-9 interacts with both PHD2 and HIF-1α, promoting hydroxylation. This step is essential to bind the von Hippel-Lindau protein (VHL) that, in turn, interacts with elongin C forming a ubiquitin ligase complex. This process leads to the proteasomal degradation of HIF-1α. Contrastingly, under hypoxia conditions, oxygen (O_2_) deprivation inhibits the hydroxylation reactions and/or increases the mitochondrial production of reactive oxygen species (ROS), which oxidize the ferrous ion in the catalytic site of hydroxylases, thereby inhibiting these enzymes. In this way, the stability and the transactivation function of HIF-1α are increased, promoting dimerization with HIF-1β. This determines the activation of the complex and the consecutive binding to the hypoxia responsive element (HRE) sequence 5′-(A/G)CGTG-3′ in target genes, increasing the transcription into mRNA (6, 7).

More than 40 different HIF-1 target genes are regulated in this way. Importantly, these genes regulate crucial cellular processes such as tumor progression, remodeling of the microenvironment, cell proliferation, survival, angiogenesis, glucose metabolism, and iron homeostasis. Proteins encoded by these genes, such as vascular endothelial growth factor (VEGF), mediate increased O_2_ delivery via the formation of new blood vessels. The expression of other HIF-1-regulated proteins, such as hexokinase II (HKII), which catalyzes the first step of glycolysis (8), glucose transporters (GLUT), and glycolytic enzymes, allow cells to adapt their metabolism to a specific environment distinguished by a reduction or lack of oxygen. Finally, the expression of a third group of gene products may influence the balance between apoptotic and anti-apoptotic signals that determine cell survival (9).

### EZH2 and its inhibitors

Enhancer of zeste homolog 2 is the catalytic subunit of the PRC2, a highly conserved histone methyltransferase, involved in the methylation of lysine 27 of histone 3.The overexpression of EZH2 has been correlated with many cancers, such as prostate, breast, bladder, whereas other kinds of cancer, such as B-cell lymphomas or other forms of breast cancer, can be promoted by specific mutations (10). For this reason, EZH2 can be considered as an interesting target for the development of targeted anticancer strategies (10).

The PRC2 complex operates as a chromatin and it is expressed in several organisms from plants to flies modifier, and humans. It interferes with the transcription of target genes (i.e., silencing genes involved in differentiation) by trimethylating lysine 27 on histone 3 (H3K27me3), considered as its major function “*in vivo*.” The human PRC2 complex is composed of five different subunits: EZH2, SUZ12, EED, AEBP2, and RbAp46/48 (10–12). In addition, EZH2, which represents the main subunit of PRC2 sequentially catalyzes three methylation reactions at H3K27, generating monomethylated, dimethylated, and trimethylated H3K27 (H3K27me1, H3K27me2, and H3K27me3). In the PRC2 complex, EZH2 represents the most important subunit, SUZ12 and EED are important for maintaining the integrity of the entire structure, while AEBP2 is necessary to stabilize the structure of the complex. Mutations of these subunits could determine a lack of PRC2 function. In contrast, the RbAp48 subunit does not seem to be necessary during the enzymatic activity, and therefore it may be not essential for the histone methyltransferase action of EZH2 (12).

**Figure 1:**
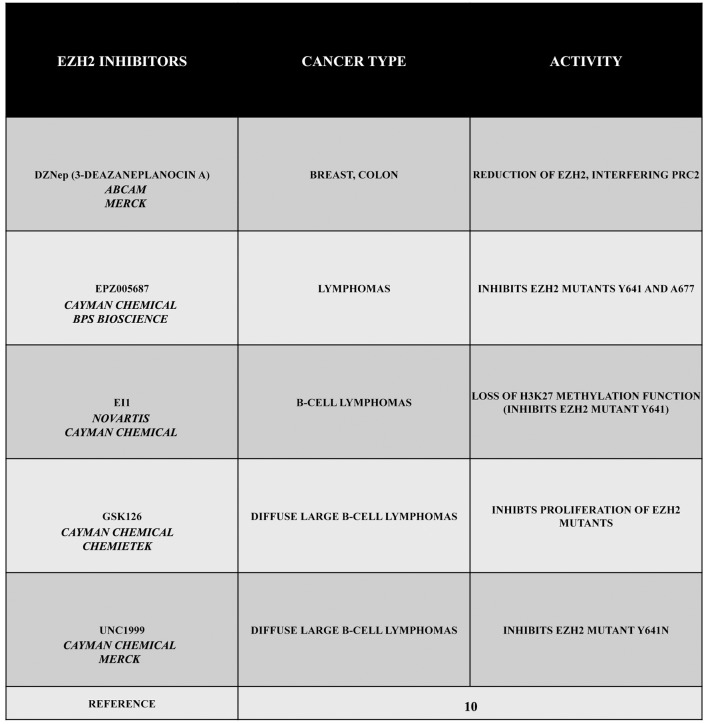
EZH2 inhibitors 3-deazaneplanocin A (DZNep), EPZ005687, EI1, GSK126, and UNC1999. DZNep is a S-adenosylhomocysteine hydrolase inhibitor, depletes EZH2 and the associated H3K27me3, and induces apoptosis in breast and colon cancer cells. EPZ005687 inhibits H3K27 methylation by the EZH2 mutants Y641 and A677, and it has been shown to selectively kill lymphoma cells that are heterozygous for one of these EZH2 mutations. EI1 is active against different forms of EZH2, while GSK126 effectively inhibits the proliferation of the protein mutants in DLBCL cell lines. UNC1999 represents the first orally bioavailable EZH2 inhibitor.

Given its specific activities, EZH2 can be considered as an interesting target in several types of cancer. For this reason, during the past years, a large number of EZH2 inhibitors have been discovered, such as 3-deazaneplanocin A (DZNep), EPZ005687, EI1, GSK126, or UNC1999 (Figure 1). Obviously, the development of these inhibitors is limited to those cancers in which there is a gain of function of EZH2. Therefore, in this section, we describe different types or tumors indicating, when possible, the state of EZH2 (wild type, overexpressed, mutated but still targetable by inhibitors or mutated with a loss of function). The reader must keep in mind that EZH2 inhibitors are useless or even harmful in those cancers characterized by a loss of function of EZH2. Nonetheless, this knowledge is important for both scientists as well as patients.

The drug DZNep is an S-adenosylhomocysteine hydrolase inhibitor that reduces EZH2 and H3K27me3 levels, thereby inducing apoptosis in breast and colon cancer cells. This compound alters the PRC2 pathway even if the exact mechanism is yet to be elucidated, but it is considered as a promising drug for future cancer therapies. The drug EPZ005687 was discovered as an inhibitor with a 50 fold higher selectivity for EZH2 than for EZH1 (10). Moreover, EPZ005687 can also inhibit H3K27 methylation induced by two EZH2 mutants (Y641 and A677), thereby killing lymphoma cells carrying the Y641 or A677 EZH2 mutant (10, 13). In addition, EI1 is active against different forms of EZH2, while the most potent inhibitor of EZH2, GSK126, is effective in inhibiting proliferation of the protein mutants in DLBCL cells (10). The drug UNC1999 an analog of GSK126, represents the first orally bioavailable EZH2 inhibitor. *In vitro* UNC1999 has shown high efficiency against wild type and mutant EZH2 against many epigenetic and non-epigenetic targets. In cells, UNC1999 seems to reduce H3K27me3 levels (IC_50_<50 nmol/L) reducing the vitality of DLBCL cell lines with the Y641N mutation (14). Recent genetic studies in a wide range of organisms have shown an evolutionarily conserved antagonistic relationship between polycomb proteins and switch/sucrose non-fermentable (SWI/SNF) complexes, which utilize the energy of ATP hydrolysis for chromatin remodeling. These complexes are composed of 12 to 15 subunits that are mutated in 20% of all human cancers. Moreover, in several cases, it has been demonstrated that EZH2 gain-of-function mutations such as in A687 and A677 can lead to cellular transformation as reported in non-Hodgkin lymphomas(15, 16). Unopposed EZH2 activity is also a driver of cancers determined by the loss of the core subunit SNF5/SMARCB1 in the SWI/SNF complex as demonstrated in rhabdoid tumor, a highly malignant aggressive type of pediatric cancer (17, 18). Additionally, EZH2 has been shown to also possess non-enzymatic functions, leading to a situation that raises the possibility that the enzymatic inhibitors currently employed in clinical trials may not fully suppress EZH2 activity as well as its tumor promotion. However, this effect may be reduced by blocking the interaction between EZH2 and other PRC2 subunits using a peptide known as *stabilized alpha-helix of EZH2* (SAH-EZH2) obtained from the EZH2 domain interacting with the EED subunit. The SAH-EZH2 interaction interferes with the EZH2–EED complex, reducing EZH2 levels, and stopping H3K27 trimethylation. This peptide is effective against EZH2-dependent MLL–AF9 leukemia and EZH2-mutant lymphoma cells, whereas it does not have any effect on non-transformed and EZH2 controls. Remarkably, the anti-proliferative effect of SAH-EZH2 has been linked more to the reduction of EZH2 expression than to H3K27me3 reduction. These results are consistent with the observations stressing the important role of the non-enzymatic function of EZH2 in SWI/SNF-mutant cancers (11).

## Hypoxia master regulator HIF-1 and its inhibitors

During recent years, a growing number of evidence has laid the groundwork for a better comprehension of all the complicated processes implicated in the development of cancers. Transforming cells are characterized by an uncontrolled growth forming the tumor mass, and in the internal core, the reduction of oxygen induces a great number of cellular modifications leading the cells to adapt to this particular microenvironment. All these hypoxia-adaptive changes include the switch from oxidative phosphorylation to glycolysis in cell metabolism, an augmented synthesis of glycogen, and a switch from glucose to glutamine as a substrate for the synthesis of fatty acids. In this particular condition, HIF-1 acts as a primary controller of a great number of proteins implicated in a wide variety of cellular activities. The presence of hypoxic regions inside the tumor results in (I) necrosis of cells distant from vessels of host tissue and (II) activation of the HIF-1 complex in an attempt to increase the survival of sublethally damaged tumor cells. In this situation, HIF-1 expression increases not only the survival of tumor cells but also their commitment to malignancy (19). In cancer cells, HIF-1 can also be activated by loss of function of the tumor suppressor VHL and by gain of function of oncogenes leading to the activation of the PI3K/AKT/mTOR pathway. Moreover, HIF-1 activation regulates the metabolism to increase cancer progression and resistance to therapy. For this reason, HIF-1 or metabolic enzyme inhibitors may be used to target the metabolic flexibility of cancer cells increasing their sensitivity to anticancer therapies (20).

During the past years, a growing number of molecules have been demonstrated to inhibit HIF-1 activity. A large number of these inhibitors work by reducing HIF-1α mRNA or protein levels, HIF-1 DNA-binding activity, or the trans-activation of some HIF-1 related genes. These drugs reduce the expression of HIF-1α operating on the synthesis or degradation of proteins. In many cancers, the synthesis of HIF-1α is strictly related to mTOR activity. In fact, in cancer cells, the continuous activation of tyrosine kinase receptors (such as HER2neu, BCR-ABL, and EGFR) and/or of the downstream phosphatidylinositol 3-kinase/AKT and RAS/MAP kinase signal transduction pathways determines the growth of mTOR activity and the consecutive activation of HIF-1. Thus, inhibitors of these pathways are able to determine the loss of HIF-1 activity and other biological activities such as a reduction of tumor vascularization promoting the therapeutic effect (21).

Moreover, HIF-1 expression can be also controlled by redox (reduction-oxidation)-dependent processes. In fact, it has been demonstrated that by treating purified HIF-1 with diamide, hydrogen peroxide, or N-ethyl-maleimide, a specific alkylator of cysteine sulfhydryl groups, it is possible to cause a complete loss of DNA binding activity (22).

On the basis of these data, it is obvious to consider HIF-1 as an important target for anticancer therapy, and during the past years, several natural products (generally, the term “natural product” is used to indicate low molecular weight secondary metabolites produced by animals, plants, and microbes for chemical defense and growth advantage) or synthetic compounds have been shown to possess an anti HIF-1 activity.

## Natural inhibitors

Several emerging scientific data show the efficacy of many natural compounds to inhibit the activity of HIF-1 operating on protein synthesis or protein degradation. Actinomycin D (dactinomycin) is a metabolite produced by *Streptomyces parvullus* (formerly *S. antibioticus*) that acts as an inhibitor of transcription, and it is able to block hypoxia-induced HIF-1 activity in the human hepatoma cell line Hep3B. Moreover, other data showed the efficacy of actinomycin D to block HIF-1α induction by angiotensin II (Ang II) in the vascular smooth muscle cells of rats without altering hypoxia induction. Inhibition of HIF-1 depended on the cell type, stimulus, and drug concentration. Another cytotoxic compound, GL331, is a semisynthetic podophyllotoxin-derived topoisomerase II inhibitor with IC_50_ values in the range of 0.5 μm to 2 μm that has been shown to work against a large number of tumor cell lines. In the human lung adenocarcinoma cell line CL1-5, using a concentration of 10 μm of GL331, it has been possible to decrease HIF-1α mRNA levels, probably through transcriptional inhibition. However, such an inhibitory effect is not specific for HIF-1. At the same concentrations, the compound shows cytotoxicity and inhibits the expression of cyclin D1 in CL1-5 cells. In addition, GL331 demonstrated no effects against gastric cancer in a clinical study (23).

Emerging data have discovered a growing number of natural products capable of reducing HIF-1α mRNA levels despite the fact that the mechanism is not completely clear for all of them. The Indian traditional medicine glycoside known as picroliv (in which the major compound found is a purified iridoid glycoside fraction picroside-I and kutkoside from the roots of *Picrorhiza kurrooa*) reduced HIF-1α and VEGF mRNA levels *in vitro* (24, 25).

Other compounds have the ability to promote HIF-1 degradation through different ways. For example, geldanamycin (GA), a metabolite of *Streptomyces hygroscopicus*, interferes with hsp90 through the amino-terminal ATP/ADP binding pocket. In addition, GA blocks the activation of the HIF-1 complex promoting the degradation of the α-subunit through a VHL-independent proteasomal mechanism both in normoxic and hypoxic conditions (26, 27).

Other emerging scientific data have shown that by using the antifungal antibiotic cycloheximide isolated from *Streptomyces griseus*, it is possible to inhibit general eukaryotic protein synthesis and to interfere with the accumulation and activation of the HIF-1α protein. Another important group of compounds with inhibitory effects on HIF-1α protein synthesis is represented by microtubule disrupting agents (MDA) (23). Additionally, other hsp90 inhibitors such as 17-N-allylamino-17demethoxygeldanamycin, resveratrol, the antibiotic novobiocin, and many others have been shown to have an activity against the accumulation of HIF-1 (23).

Finally, another natural compound with an anti HIF-1 activity is the red Korean ginseng, even if the mechanisms of action and the antitumor effect are yet to be clarified (19, 28).

**Figure 2:**
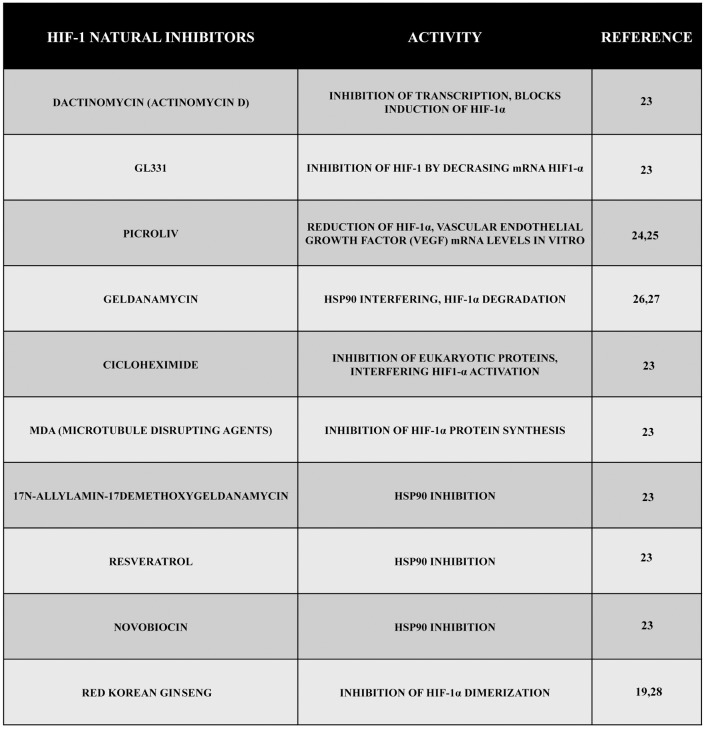
Natural inhibitors of HIF-1. During these years, a large number of compounds have been tested, and their natural inhibitory effect during hypoxia has been demonstrated. Here, we reported some of them presenting different mechanisms of action such as inhibition of transcription, inhibition of VEGF, inhibition of HIF-1 synthesis or dimerization, etc.

Therefore, as reported in this review, there are several natural drugs and compounds capable of interfering with HIF-1 activity, accumulation, and function, and this can possibly lead to the development of new anticancer strategies in the future (Figure 2). However, even if a growing number of evidence is collected day by day, still several sets of data are necessarily required to go on in this way.

## HIF–EZH2: two sides of the same coin?

**Figure 3:**
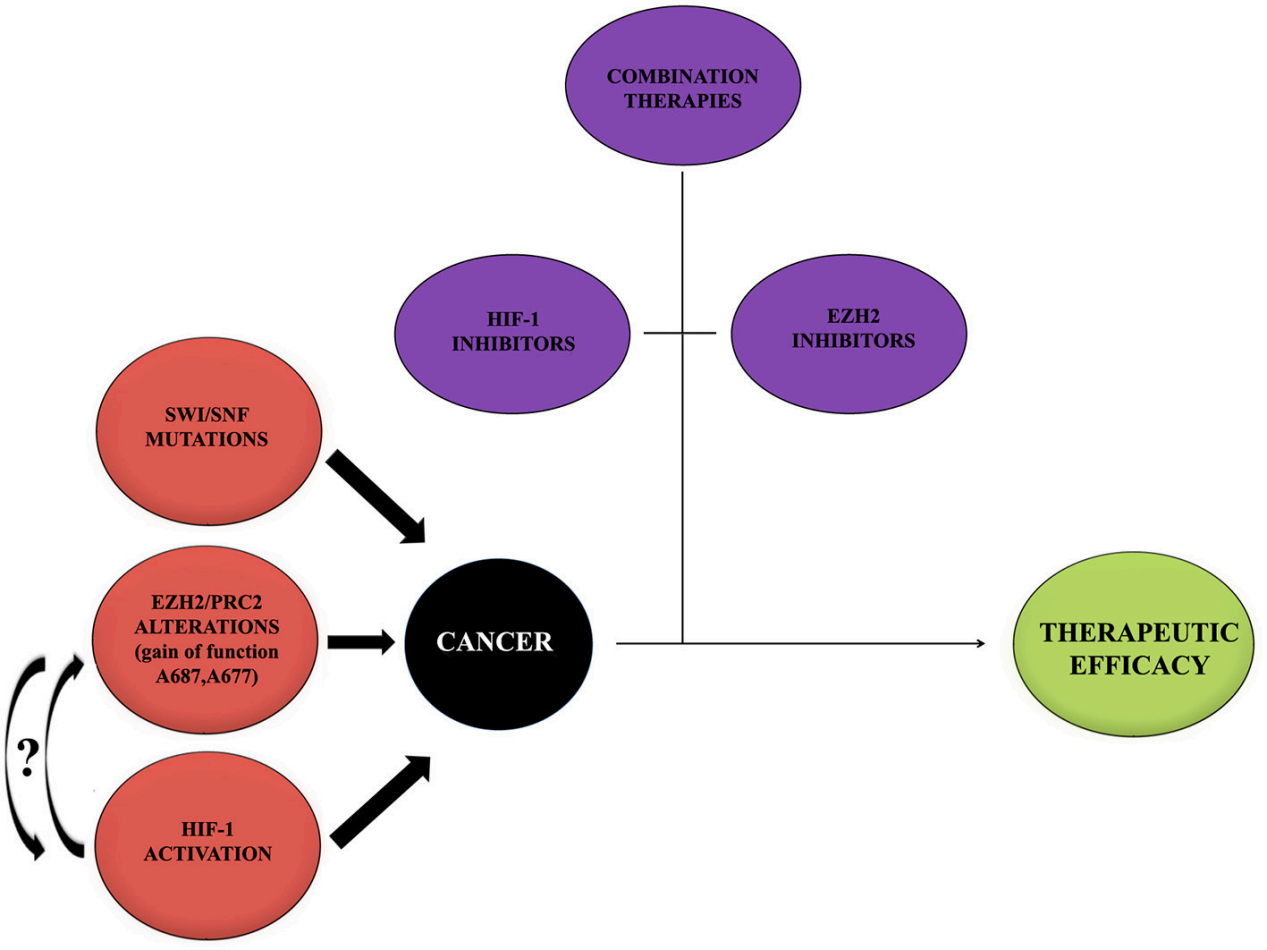
Cancer onset influencing mediators. Cancer represents a multifactorial pathology in which a large number of alterations act to promote the onset and development. Alterations of EZH2, HIF-1, and mutations of SWI/SNF may provide a negative influence promoting tumors. Curved arrows with question marks refer to the fact that no data or very little data are available regarding the possible interaction between EZH2 and HIF-1 during cancer onset. On the contrary, thanks to recent studies, the importance of single treatments with either HIF-1 or EZH2 inhibitors, combined with other therapeutic approaches, to contrast the tumor and to obtain a therapeutic efficacy has been shown. It would be interesting to try a new approach with inhibitors for both EZH2 and HIF-1.

During the past years, great progresses have been made in many areas of pediatric oncology even if tumors of the central nervous system (CNS) remain a significant challenge because of their complicated nature. Basing on recent scientific data, it has been possible to achieve a better comprehension of cancer molecular mechanisms and to develop new therapeutic approaches focused on different molecules and pathways (Figure 3).

In this review, we paid particular attention on some specific pediatric tumors such as high grade glioma and pediatric low grade glioma.

Pediatric high grade gliomas (pHGGs) have the same histological, but not molecular, features of adult HGGs (aHGGs) and have been classified, in accordance with the World Health Organization (WHO), as grade III and IV CNS tumors. Grade III glioma (anaplastic astrocytoma) is characterized, histologically, by atypical nuclei, an increased cellularity, and an increased mitotic activity. Grade IV glioma, also known as glioblastoma multiforme (GBM), is the most pathologically advanced and clinically aggressive tumor. A wide number of genomic analyses on several pediatric tumors such as glioblastomas, anaplastic astrocytomas, and diffuse intrinsic pontine gliomas (DIPG) have revealed frequent mutations of the *H3F3A* gene (29) (encoding H3.3) in which the lysine residue at position 27 was substituted with a methionine (K27M) or the glycine residue at position 34 with an arginine or valine (G34R/V) (30–33). Conversely, just a limited number of DIPGs revealed a replacement of the lysine residue at position 27 with a methionine (K27M) in the *HIST1H3B* gene encoding for the histone H3.1 (33). In fact, pediatric GBM with *H3F3A* K27M mutations shows a reduction in H3K27me3 when compared with wild type tumors. Moreover, anaplastic astrocytomas also revealed the presence of a significant fraction of hypoxic tissue areas. In fact, hypoxia has been shown to play an important role in the malignant progression of various tumor entities, and hypoxic areas have been found in malignancies such as breast, prostate, pancreas, and lung cancer, soft tissue sarcomas, non-Hodgkin's lymphomas, melanomas, liver tumors, etc (34). Hypoxia leads to the activation of HIF-1 via the stabilization of its α-subunit. Importantly, a number of studies have indicated a widespread expression of HIF-1α (35–39) and its target genes glucose transporter (GLUT)-1 (40) and carbonic anhydrase (CA) IX (40, 41), in malignant astrocytomas. Hypoxia has been suggested as an adverse prognostic factor for patient outcome. In fact, several studies of tumor hypoxia involving the direct assessment of the oxygenation status have suggested worse disease-free survival for patients with hypoxic cervical cancers or soft tissue sarcomas. In head & neck cancers, studies suggest that hypoxia is prognostic for survival (34). In fact, the identification and quantification of hypoxic regions is extremely important for the management of the disease. A large number of predictive assays for tumor oxygenation status have been developed in the past years showing differences in the degrees of success. To date, functional imaging techniques based on positron emission tomography (PET) have been demonstrated to be fundamental for both pretreatment and tumor response evaluation during therapy. Several hypoxia-specific PET markers have been developed in several clinics to quantify hypoxic tumor sub-volumes for personalized treatment planning. Moreover, a large number of new radiotracers are actually under investigation. In addition, PET-derived functional parameters and tracer pharmacokinetics can be used to obtain very useful input data to create computational models aimed to simulate or interpret PET acquired data to develop new treatment planning or radio/chemotherapy response prediction programs (42).

Primary GBM is characterized by increased vascular proliferation and necrosis in addition to the features of grade III glioma (43). In particular, pHGGs have a specific wide distribution in contrast to aHGGs that are more frequent in the cerebral cortex (44). Molecularly, pHGGs are distinct from aHGG and are characterized by a great number of activating or inactivating gene modifications, such as gene amplifications, deletions, and inactivation of p53 and histone 3.3 (H3F3A), causing them to be activated and inactivated (45).

The canonical treatment for pHGG occurring in the cerebrum typically includes initial surgery followed by radiation and chemotherapy. There is widespread agreement that total resection of tumor tissue improves patient outcome, but anyway, focal radiation therapy has also become standard in the treatment of patients greater than 3 years of age with pHGG (43). Chemotherapy is frequently used to treat patients with pHGG; although evidence of efficacy is modest, chemotherapeutic agents are often employed in the treatment of these patients in combination with temozolomide even if the ability of this drug is still debated (46–48).

Medulloblastoma (MB) is another pediatric brain tumor, representing 15–20% of the tumors occurring at a young age. While it can occur at any age from infancy through adulthood, it is most typically seen in children with bimodal incidence peaks between 3 and 4 or 8 and 9 years of age (49). MB is characterized by four distinct molecular subgroups, such as those dependent on Wingless (WNT) or Sonic Hedgehog (SHH) signaling and those that are less well characterized (Group 3 (G3) and Group 4 (G4)), showing multiple responses to common therapeutic approaches (50). These tumors present, in contrast to most other cancers, some recurrent mutations. These four MB subgroups have different mutational landscape, gene expression, pathology, and prognosis (50–54). In fact, about 25% of MBs are G3 tumors with high MYC levels as a result of somatic MYC gene amplification in about 15–20% of G3 cases. Moreover, some G3 MBs have been associated with relatively high levels of EZH2 and to increased global H3K27me3 chromatin repressive marks (53). In contrast, the H3K27me3-specific demethylase, KDM6A/UTX, is mutated in a reciprocally exclusive manner for the most part in G4 MBs (53, 55–57). Basing on this knowledge, recently, it has been possible to investigate the relationship between G3 MB and EZH2 and to explore its functional relationship with other genes that are important for the development of MB, using gene editing systems. In fact, in this way, it has been possible to demonstrate that the deletion of EZH2 accelerates de novo MB development and the progression of established MBs and that EZH2 inactivation in G3 MB implicates Gfi1 (a proto-oncogene frequently activated in human G3 MBs whose disruption antagonizes the tumor promoting effects of Ezh2 loss) as an oncogenic target gene. Therefore, enforced expression of Gfi1 promotes secondary G3 MB formation mimicking the effects of the absence of Ezh2, whereas the lack of Gfi1contrasted the effects of Ezh2 inactivation, together providing at least a partial rationale for Ezh2-mediated tumor suppression (58). Importantly, these new results point the attention to the complexity of the pathways directly and indirectly influenced by EZH2 to the point that this protein can function as either a tumor suppressor or promoter depending on the cellular context.

Ependymomas occur in both children and adults and can arise throughout the entire neuraxis. They are divided, according to the WHO, into three types classified as grade I, II, and III. While spinal cord tumors are more common in adults, in pediatric patients, approximately 70% of ependymomas arise in the posterior fossa. The canonical treatment for these specific tumors is based mainly on surgical resection, and in the post-operative period, radiation can be used to improve the survival frequency (49).

During the pediatric age, other kinds of tumor such as low grade glioma (PLGG) can cause several problems to patients. This kind of tumor represents the most common type of pediatric astrocytoma and pediatric brain tumor in general. According to the WHO classification, PLGGs are grade I or II and include pilocytic astrocytoma (PA), subependymal giant cell astrocytoma (SEGA), pilomyxoid astrocytoma (PMA), pleomorphic xanthoastrocytoma (PXA), and low-grade fibrillary astrocytoma or diffuse astrocytoma. Among these tumors, PA is considered the most common glioma in children.

Actually, these tumors are treated using different drugs including carboplatin, vincristine, temozolomide, etc. Anyway, surgical resection is commonly used to treat subependymal giant cell astrocytoma especially in the case of intracranial hypertension.

Apart from pediatric brain tumors, epigenetic alterations have an important role in several cancers; for example in leukemias, repressive proteins of the polycomb group, PRC1 and PRC2, are the main epigenetic regulators in developmental and transcriptional repression.

For example, early T cell precursor acute lymphoblastic leukemia (ETP-ALL) represents an aggressive subtype of ALL characterized by the presence of stem cells and transcriptional programs observed in myeloid cells (59). It has been demonstrated that inactivating alterations of the PRC2 components are recurrent in this kind of human tumor, but their functional role is yet to be entirely defined (60). Some data about the involvement of EZH2 have been collected using a murine model of *NRASQ61K*-driven leukemia that shows the phenotypic and transcriptional aspects of ETP-ALL (60). In particular, homozygous inactivation of EZH2 cooperates with oncogenic NRASQ61K to favor leukemia development. In addition, EZH2 acts as silencer of stem cell- and early progenitor cell-associated genes, and its loss is associated with an increased activation of signal transducer and activator of transcription 3 (STAT3) by tyrosine 705 phosphorylation (60). Moreover, the activity of STAT3 maintains the co-option of a pro-metastatic program in activated astrocytes in a metastatic lesion. These astrocytes support metastatic cells through their ability to modulate the innate and acquired immune systems, and it has been demonstrated that patients who have reactive astrocytes with active STAT3, show a reduced survival from the diagnosis of intracranial metastases. In fact, if in reactive astrocytes the signaling of STAT3 is prevented, it is possible to reduce the formation of metastasis in the brain from different primary tumor sources. Thus, by using an orally bioavailable treatment against STAT3, it is possible to obtain an antitumor effect in patients even with advanced systemic diseases such as brain metastasis (61).

Therefore, according to these data, the inactivation of EZH2 can be linked to the activation of transcriptional programs in stem cells and to increased growth/survival signaling, characteristics determining a poor prognosis in patients (60).

T-cell acute lymphoblastic leukemia (T-ALL) represents about 10–15 % of pediatric ALL cases and, when compared with B-progenitor ALL patients, shows some aggressive clinical features such as higher risk for primary resistant disease, and frequent early isolated central nervous system relapse. In recent years, thanks to different therapeutic strategies based on intensive chemotherapy, T-ALL prognosis in children and adolescents has improved. However, it is still worse when compared with B-lineage ALL in particular if associated to a lack of initial response to the therapy with prednisone (62). Another approach to treat this tumor is based on 3-deazaneplanocin-A (DZNep) blocking EZH2. This agent showed a good activity inducing a strong apoptosis of cancer cells; however, additional data have to be collected regarding its potential for treating T-cell ALL (63).

Other therapeutic approaches for the treatment of this cancer are based on daunoblastine, an anti-leukemic agent used for decades despite the fact that its success is frequently linked to the use of other drugs. For this reason, recently, the efficacy of this drug in combination with DZNep has been shown. A combination of these drugs in the ratio 25:50 showed a synergistic effect at the CalcuSyn elaboration (a software to explore the relative contribution of each agent to the synergism) and induced apoptosis in Jurkat cells. In particular, DZNep caused 63 % of apoptosis if used alone, whereas a stronger effect up to the 67% was observed if combined with daunoblastine after 72 h of treatment (64). Therefore, DZNep inhibits Jurkat cell proliferation through the activation of check-points that block cell cycle arrest at S phase, an effect that results in the induction of apoptosis. In fact, EZH2 was upregulated in natural killer/T-cell lymphoma, through Myc-mediated mRNA inhibition. Such EZH2 upregulation determines the activation of cyclin D transcription promoting cell proliferation, an effect that is independent of its methyltransferase activity (65).

Even if the principal activity of EZH2 as a gene silencer proceeds through the methylation of H3K27, a large number of studies have shown that the transcriptional activation of EZH2 in many types of cancer is achieved independently of H3K27me (66). This activity has been demonstrated especially in natural killer/T-cell lymphoma, in which cyclin D transcription is activated by EZH2 upregulation via Myc-mediated mRNA inhibition promoting cell proliferation without a methyltransferase activity (65). Similarly, in Jurkat cells, when EZH2 is upregulated, there is an activation of cyclin D transcription demonstrating the effect of DZNep. The data obtained showed that using both agents causes a change in the expression and activity of several central proteins such as caspase-3, 9, Bcl-2, Erk, and EZH2. Specifically, a reduction of Bcl-2 and an increase of cleaved caspase-3 and caspase-9 especially in cells treated with therapy combination has been observed. Moreover, the combination also inhibited Erk-mediated proliferation pathway. This observation has been considered extremely important since it gives the possibility to develop new therapeutic approaches based on the combination of DZNep and daunoblastine to treat T-ALL cells, in particular the subgroup with a much worse prognosis (63).

Along with PRC2, another main factor promoting the development and progression of cancer is represented by the microenvironment and its main responder HIF even for what concerns pediatric tumors. In fact, several studies reported a large number of brain and non-brain tumors in which HIF plays a primary role (39, 67–69). One example is that of metformin, a well-known insulin-sensitizer frequently used for type 2 diabetes therapy, which can be potentially considered as a very interesting drug in oncology also. In fact, the use of metformin has been hypothesized to contrast pediatric sarcomas such as osteosarcoma, Ewing sarcoma, and rhabdomyosarcoma, representing common pediatric sarcomas, that depend on insulin-like growth factor (IGF) and insulin for pathogenesis and progression. *In vitro* results showed antiproliferative and chemosensitizing effects, but these results have not been confirmed in *in vivo* experiments related to Ewing sarcoma even if combined with vincristine. It is suggested that hypoxia, present in solid tumors, may be the cause of the discrepancy between *in vitro* and *in vivo* effects. In fact, some evidence confirmed that hypoxia can prevent the antitumoral mechanism of action of metformin, that is, activation of AMPK and inhibition of mTOR signaling. Thus, the use of metformin for conventional chemotherapy may be limited to low hypoxic tumors. For this reason, the influence of hypoxia should be always considered when using novel therapies to treat these sarcomas in particular (67).

As frequently demonstrated, the development and progression of a tumor is linked to a large number of molecules and/or to a long sequence of events and modifications leading to the proliferation of cells. The tumor suppressor gene p53 and its family members p63/p73 are the main regulators of the cell cycle and critical determinants of tumorigenesis. ΔNp63 is a splice variant of p63, which lacks the N-terminal transactivation domain and acts as an antagonist of p53-, p63-, and p73- dependent translation, contrasting their tumor suppressor activity. Recent studies of pediatric neuroblastoma and osteosarcoma, demonstrated an increased expression of 1Np63, but it has not been possible to correlate ΔNp63 expression with p53 mutation status (70). For this reason, a possible mechanism leading ΔNp63 itself to favor the cells' malignant transformation with a gain of function independently by any p53 antagonism has been supposed. The overexpression of ΔNp63, independent of p53, increases the secretion of interleukin-6 (IL-6) and interleukin-8 (IL-8), leading to the elevated phosphorylation of STAT3 (Tyr-705). This condition leads to the stabilization of the HIF-1α protein, resulting in VEGF secretion. These data suggest that high levels of ΔNp63α are expressed in pediatric neuroblastoma and osteosarcoma, ΔNp63α has oncogenic effects in neuroblastoma and osteosarcoma, ΔNp63α regulates VEGF activity and promotes migration, induces STAT3 phosphorylation, and increases the transcription of interleukin (IL-6 and IL-8), and that ΔNp63 expression is enhanced in osteosarcoma lung metastases (70).

As reported for many kinds of tumor, hypoxia plays a primary role in the development, and progression of the pathology, influencing the specific microenvironment in which tumor cells live. One of the most dangerous but also uncommon tumors with <50 cases reported in literature (71, 72), with a poor cure of approximately 2 months (72) in infancy even with intensive therapy, is congenital glioblastoma (cGBM). The prognosis of this tumor is absolutely poor, but surgery and/or chemotherapy can increase long-term survival (24–33 months) (72) if the infants survive birth and initial surgery or biopsy. Histologically, they are very similar to other GBMs and present themselves with some typical features such as hypercellularity, high ability to infiltrate in the glia with necrosis and/or pseudopalisading necrosis, vascular proliferation, and increased mitotic activity and MIB-1 rate (72, 73). Following these observations, recently, it has been carried in a study on five patients with congenital GBMs in the first 3 months of life. Glioblastomas were situated in the deep gray matter close to the ventricles, and three of the patients had an extension of the tumor parenchyma into the intraventricular space. Four patients survived surgery, but the tumor was completely removed only in one of them. Cytogenetic studies on one patient demonstrated the presence of a diploid population; meanwhile, in two of the three patients, a diploid clone was observed in combination with aneuploidy in a near tetraploid context, while in the other, tetraploidy was seen in combination with diploidy. Furthermore, another interesting observation of this study is the difference in ploidy between this tumor and the small cell astrocytoma/GBM. However, FISH analysis revealed that this tumor type possesses disseminated diploid populations (74). Hierarchical clustering analysis in the three cGBMs has shown a similar gene expression also comparable to other high-grade adult and pediatric gliomas. Comparing cGBMs with pediatric and adult GBMs revealed that only 31 genes were differentially expressed. This can be considered to be, despite the different scale, very similar to the differences observed between pediatric GBMs and adult GBMs. Furthermore, in all three cGBM samples measured, EGFR gene expression was very low, and additionally amplification in EGFR was not observed, a result very similar to pediatric GBMs. Moreover, functional analysis showed that more that 50% of the differing genes had a role in signal transduction, including four receptor tyrosine kinases, and 39% of the differing genes in cGBMS, were involved in glucose metabolism. Thanks to these evidence, it has been possible to differentiate cGBMs from pediatric and adult GBMs. Furthermore, in some cases, cGBMs may have a better prognosis than pediatric or adult GBMs, however, this is strongly dependent on the ability of the infants to survive birth, tolerate surgery, and undergo chemotherapy. Patients who were exposed to surgery with subtotal resection or biopsy also did well, suggesting that it is not necessary to base the therapeutic approach on aggressive surgery probably inducing more morbidity in this population. Since these tumors are characterized by high vascularization and bleeding, patients with cGBMs show a good response to moderately intense chemotherapy regimens without requiring stem cell rescue or dose-dense chemotherapy to obtain a response. In conclusion, this study provides important information on a rare tumor. Unfortunately, such information is limited due to the small sample size, and for this reason, additional evidence is necessary to solidify both the molecular and chemotherapeutic results (75).

## HIF–EZH2 connection

The correlation between HIF-1 and EZH2 is an interesting but yet uncharted aspect of tumor biology. There are few scientific studies connecting hypoxia, HIF-1, and EZH2 during tumor formation or tumor progression. The majority of such studies concord with the idea that hypoxia in general and HIF-1 in particular increase EZH2 expression. In fact, in pancreatic cancer cells, hypoxia stabilizes HIF-1α that, in turn, increases TWIST expression. TWIST accumulation increases EZH2 expression and tumorigenesis measured as epithelial-mesenchimal transition (EMT) and xenograft growth (76). Similarly, in hepatocellular carcinoma (HCC), hypoxia and HIF-1α increases NIPP and EZH2 levels, an effect that then results in augmented invasive and metastatic potential (77). Interestingly, abrogation of NIPP and EZH2 expression, prevented such a malignant phenotype (77).

Another example regarding the correlation between HIF-1 and EZH2 has been revealed in a study conducted some years ago in which the expression of EZH2 in breast tumor initiating cells (BTICs) is enhanced by hypoxia through HIF1α-mediated transactivation, which promotes the expansion of BTICs and cancer progression (78, 79). The enhanced expression of EZH2 in BTICs determines the downregulation of DNA damage repair proteins and the accumulation of genomic abnormalities that mediate deregulated signaling (RAF1-ERK-β-catenin) resulting in BTIC expansion and cancer progression. Moreover, it has been revealed that the cancer-predisposed hypoxic microenvironment may promote BTICs through the upregulation of EZH2 expression. Furthermore, a specific genomic aberration mediated by EZH2-impaired DNA damage response has been linked to the expansion of BTICs and, finally, it has been possible to show a previously unidentified therapeutic effect of the inhibitors of RAF1-ERK signaling (e.g., AZD6244, a specific MEK/ERK inhibitor, already tested in multiple clinical trials) to prevent breast cancer progression by eliminating BTICs with important clinical implications (79).

Interestingly, in colorectal cancer, HIF activation is achieved through the overexpression of the type II Na/Pi co-transporter SLC34A2 causing ROS accumulation. In fact, increased ROS stabilize the HIF-1α protein inducing EZH2 overexpression. The silencing of SLC34A2 completely prevented EZH2 overexpression, as well as colorectal cell proliferation and chemo-resistance (80). Another mechanism through which HIF-1α can increase EZH2 expression has been demonstrated in prostate cancer cells. In these studies, authors show that HIF-1α activation during hypoxia is accompanied by a decrease in the expression of miR-101 and the increase of EZH2 (81). In fact, normally, expression of miR-101 inhibits EZH2 production and the invasiveness of prostate cancer cells (81). Upon HIF activation, however, miR-101 levels drop, thereby increasing EZH2 expression and the invasion of prostate tumor cells (81). However, the study of the HIF-EZH2 interplay is complicated by the observation that, in glioblastoma, EZH2 activates HIF-1α expression by inhibiting the eleven-nineteen lysine-rich leukemia associated factor 2 (EAF2) that, in the prostate, regulates transcriptional elongation of RNA Poll II (82). In fact, EAF2 binds and stabilizes VHL, thereby reducing HIF-1α levels. Therefore, by decreasing EAF2 expression, EZH2 reduces VHL activity stabilizing HIF-1α expression, which is an effect that, in turn, increases tumor growth and metabolism favoring glycolysis (82).

In addition, in multiple myeloma, EZH2 inactivation upon phosphorylation is observed during the acquisition of multi drug resistance. Interestingly, in this tumor, EZH2 inactivation is accompanied by increased expression of HIF-1α and anti-apoptotic genes, an effect that then causes multidrug resistance (83).

Finally, it has been demonstrated that, under hypoxia, HIF-1α regulates the switch from the tumor suppressive to the oncogenic function of EZH2. This interesting study tries, for the first time, to reconcile these two apparently opposing effects of EZH2 by showing that EZH2 has a tumor suppressive function when part of the PRC2 and a tumor promoting function when bound to Forkhead box M1 (FoxM1) by increasing matrix metalloproteinase expression and tumor cell invasion. Importantly, HIF-1α regulates the formation of these complexes by shifting EZH2 from PRC2 to FoxM1. Such an effect is achieved through an HIF-1α-mediated decrease in the expression of the PRC2 complex (84). In conclusion, even if scientific evidence correlates that HIF-1 and EZH2 are accumulating, still there is a need to obtain more additional data before we can completely elucidate and connect the mechanisms involving both HIF-1 and EZH2 in a tumor specific context.

## Something for the future

During recent years, a large number of scientific studies revealed the importance of therapeutic targeting of PRC2. Such a consideration emerged when it was observed that EZH2 overexpression or mutations increasing EZH2 activity could initiate, promote, or maintain oncogenesis. Some of the molecules that have been already developed such as the small-molecule inhibitors of PRC2 or EZH2 are actually under clinical evaluation for the treatment of some kinds of cancers including germinal-center B-cell lymphomas characterized by EZH2 mutations, which represent about 15–20% of all cases. PRC2, or more precisely its subunit EZH2, has been implicated in the development of various hematopoietic drugs. Furthermore, some drugs that have already been developed could be used to develop more efficient therapeutic approaches and personalized therapies.

Basing on this knowledge, it is important to consider the wide potential of these new generation of drugs not only for the actual but also for future therapeutic strategies. However, caution must be taken when planning to use EZH2 inhibitors and in interpreting the results. In fact, as recently demonstrated, our knowledge of the role of EZH2 as a tumor suppressor or tumor promoter is still too limited, and new players and interlinked pathways must be detailed and studied before we can provide a valid antitumor strategy that uses inhibitors of EZH2. Moreover, the use of such inhibitors will be context-specific according to the role of EZH2 in that specific tumor.

As reported in this review, HIF-1 plays a central role in tumor growth/progression. In fact, numerous *in vitro/in vivo* experiments have demonstrated an inhibition of tumor growth elicited by many HIF-1 inhibitors at the point to activate clinical trials for some of them. In particular, 2ME2 and other derived-molecules are undergoing phase I and II clinical trials in patients with breast, prostate, and ovarian cancer (83, 84). Other drugs such as analogs of GA are employed to treat patients with VHL disease, breast cancer, etc. in phase II clinical trials (85, 86). In addition, bortezomib has been approved by the FDA for multiple myeloma treatment, based on the results from the phase II trials. Finally, EZN-2968 is currently in phase I clinical trials (85, 86).

As reported in this review and depicted in Figure 3, both EZH2 and HIF-1 play major roles in the regulation of cellular activities, and interference in their normal functions may alter the cellular homeostasis resulting in the development of different pathologies such as cancer. Even if little is known, new experiments that aim to unravel the connection between EZH2 and HIF may lay the groundwork for a better comprehension of the pathologic molecular mechanisms interlinking these two molecular players and will provide the possibility to develop, day by day, an increasing number of specific drugs to use in personalized therapies against cancer.

## Author contributions

All authors listed have made a substantial, direct and intellectual contribution to the work, and approved it for publication.

### Conflict of interest statement

The authors declare that the research was conducted in the absence of any commercial or financial relationships that could be construed as a potential conflict of interest.
